# Wnt antagonist FRZB is a muscle biomarker of denervation atrophy in amyotrophic lateral sclerosis

**DOI:** 10.1038/s41598-020-73845-z

**Published:** 2020-10-07

**Authors:** Thaddaeus Kwan, Mohamed Kazamel, Kristina Thoenes, Ying Si, Nan Jiang, Peter H. King

**Affiliations:** 1 Department of Neurology, University of Alabama at Birmingham, Civitan 545C, 1530 3rd Avenue South, Birmingham, AL 35294-0017 USA; 2 Department of Cell, Developmental, and Integrative Biology, University of Alabama at Birmingham, Birmingham, AL 35294 USA; 3 Birmingham Veterans Affairs Medical Center, Birmingham, AL 35294 USA

**Keywords:** Neurodegeneration, Amyotrophic lateral sclerosis, Neuromuscular disease, Peripheral nervous system

## Abstract

Skeletal muscle and the neuromuscular junction are the earliest sites to manifest pathological changes in amyotrophic lateral sclerosis (ALS). Based on prior studies, we have identified a molecular signature in muscle that develops early in ALS and parallels disease progression. This signature represents an intersection of signaling pathways including Smads, TGF-β, and vitamin D. Here, we show that the Wnt antagonist, Frizzled Related Protein (FRZB), was increased in ALS muscle samples and to a variable extent other denervating disease but only minimally in acquired myopathies. In the SOD1^G93A^ mouse, FRZB was upregulated in the early stages of disease (between 40 and 60 days) until end-stage. By immunohistochemistry, FRZB was predominantly localized to endomysial connective tissue and to a lesser extent muscle membrane. There was a significant increase in immunoreactivity surrounding atrophied myofibers. Because FRZB is a Wnt antagonist, we assessed β-catenin, the canonical transducer of Wnt signaling, and found increased levels mainly at the muscle membrane. In summary, we show that FRZB is part of a molecular signature of muscle denervation that may reflect disease progression in ALS. Our findings open up avenues for future investigation as to what roles FRZB and Wnt signaling might be playing in muscle denervation/reinnervation.

## Introduction

Amyotrophic lateral sclerosis (ALS) is a neurodegenerative disease whereby motor neuron degeneration results in muscle denervation and atrophy. The disease is progressive, ultimately causing respiratory failure from paralysis of the diaphragm. ALS affects approximately 5 out of every 100,000 individuals in the United States and the outlook for survival from onset is only 2 to 5 years^1,2^. Aside from familial ALS, which accounts for approximately 10% of cases, the etiology of sporadic ALS remains unclear, with numerous environmental factors potentially contributing to the development of disease^3^. This heterogeneity is further confounded by variable sites of onset and often poorly predictable rates of disease progression. This complexity leads to delays in diagnosis (average of 9 to 12 months from symptom onset) and hampers clinical trials looking at therapeutic interventions^3,4^.

Biomarker discovery remains an important research direction in ALS to help diagnose and track this complex disease in the clinic, provide insight into pathophysiological mechanisms, and possibly identify new therapeutic targets^4,5^. We have focused on skeletal muscle for ALS biomarker discovery since the earliest pathological changes occur at the neuromuscular junction (NMJ) and the myofiber^6,7^. Using transcriptome analysis of muscle biopsies from ALS patients, we previously identified a signature of upregulated molecular pathways including Smads, TGF-β, CYP27B1 and a subset of myomiRs that starts in the early stages of disease based on correlative studies with the SOD1^G93A^ mouse model^8–11^. In this report, we analyze another target identified in that analysis: the Wnt antagonist, Frizzled Related Protein (FRZB). We describe for the first time an upregulation of FRZB in muscle samples from patients with ALS and to a lesser extent other muscle-denervating diseases. Immunohistochemical analysis indicated a significantly increased association of FRZB immunoreactivity with atrophic fibers. In the SOD^G93A^ mouse, *FRZB* mRNA expression was increased in skeletal muscle beginning in early pre-symptomatic time period. Since FRZB antagonizes Wnt signaling through competitive binding to Wnt ligands^12,13^, we investigated the downstream signal transducer, β-catenin, and observed strikingly increased immunoreactivity in myofibers of ALS muscle sections, predominantly localized to the muscle membrane. Our report provides novel insight into the molecular underpinnings of muscle denervation in ALS and other denervating disease, and opens up avenues for future investigation as to whether FRZB and Wnt signaling represent therapeutic targets.

## Results

### FRZB is upregulated in ALS muscle tissue

**Figure 1:**
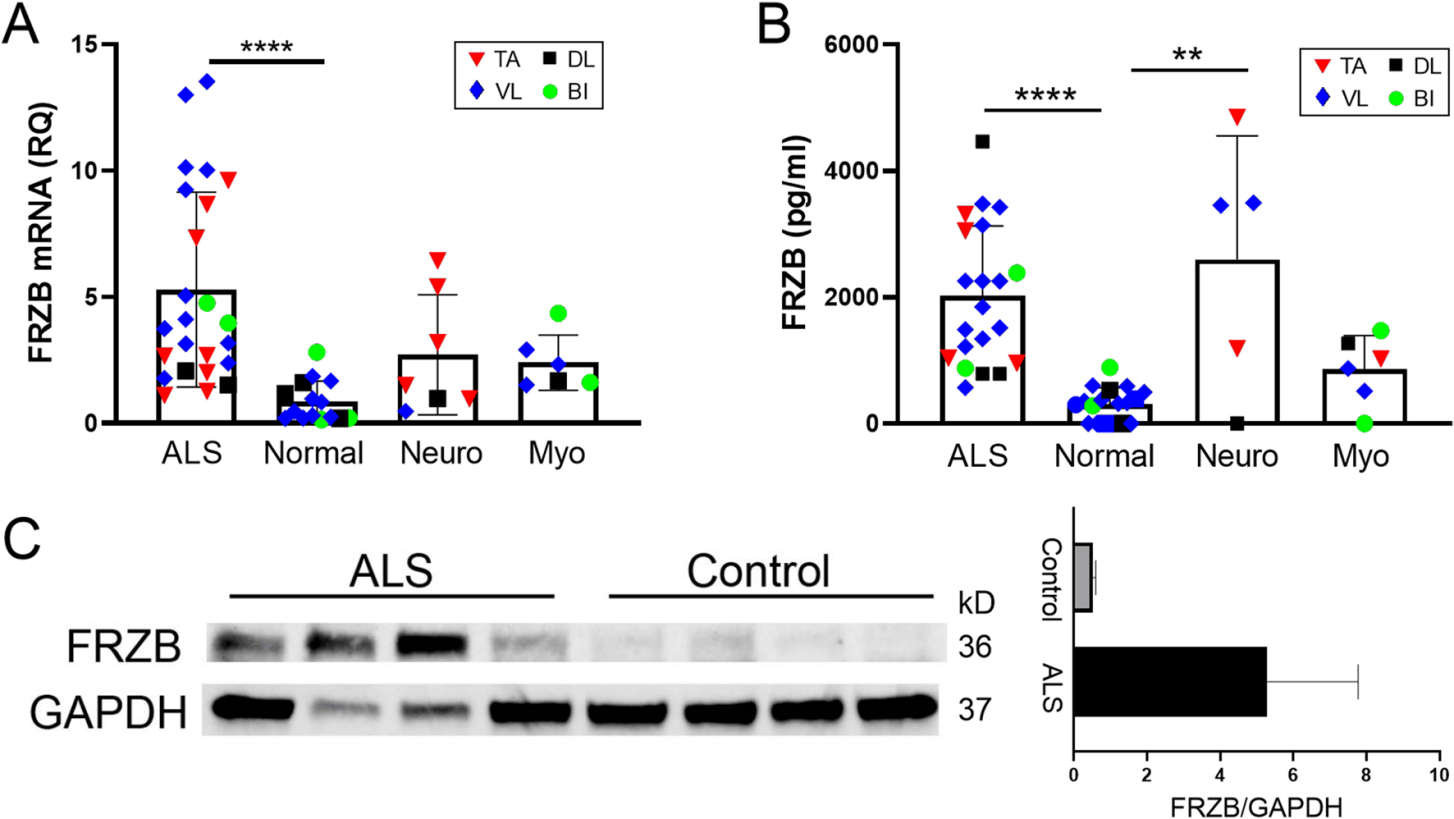
FRZB is upregulated in ALS muscle tissues. (**A**) FRZB mRNA expression in muscle was quantitated relative to GAPDH endogenous control using qPCR. Mean FRZB mRNA expression in normal control samples (n = 15) was set to 1. Mean values (± SD) for FRZB mRNA in ALS (n = 24), myopathy (n = 6), and neuropathy (n = 7) were expressed as fold-change over normal control. ****P < 0.0001. (**B**) FRZB protein expression was quantitated in muscle lysates using ELISA. Mean (± SD) FRZB protein concentrations (pg/ml) for normal control (n = 18), ALS (n = 21), myopathy (n = 6), and neuropathy (n = 5) are shown. *P < 0.5; ****P < 0.0001. (**C**) Left panel: western blot comparing FRZB expression in vastus lateralis muscle samples from ALS and normal control subjects using GAPDH as a loading control. Full blots are shown in Supplemental Fig. S1. Right panel: Quantitation (mean ± SD) of FRZB protein levels estimated by densitometry and normalized to GAPDH.

FRZB was first identified in an RNA sequencing project comparing transcriptomes between normal and ALS patients using small sample numbers^9^. To validate this finding, we expanded our sample number and included disease controls. The majority of these samples, as summarized in Table 1, were from muscle biopsies or post-mortem tissue collection of patients seen in our clinics. The demographics of our ALS population, including age, gender ratio, and site of onset (limb versus bulbar) were consistent with those we and others have reported previously^14,15^. The ages of normal subjects and disease controls were similar, although the neuropathy group had a higher number of male subjects whereas the myopathy group had a higher number of female subjects. With these samples, we first assessed *FRZB* mRNA levels by qPCR and found a 5.5-fold increase in ALS muscle samples compared to those from normal subjects (P < 0.0001; Fig. 1A). The values within the group were variable, going as high as 16-fold over the mean for the normal samples. Values for the neuropathy and myopathy cohorts were increased compared to controls, but the differences were not significant. There was considerable variance in the neuropathy values, and it is possible that the lack of significance stems from the relatively lower sample number. Alternatively, this may relate to variable severity of the neuropathic changes seen on the biopsy either due to the underlying disease or biopsy sampling. Although there is no formal or uniform grading system for severity of denervation in muscle, many of the neuropathy patients with the lowest FRZB levels were interpreted as having only mild denervation changes in the pathology report. We next measured protein levels in muscle lysates by ELISA (Fig. 1B). The ALS group had a ~ sevenfold increase in FRZB over the normal control group with all samples being increased (P < 0.0001; Fig. 1B). The neuropathy group had a similar mean fold-increase in FRZB, but similar to the mRNA analysis, there was high variance with some samples below the mean for the normal control group. The myopathy group, on the other hand, had only a small but non-significant increase over the normal controls and was significantly lower than the ALS group (P < 0.05). Western blot of ALS samples confirmed the ELISA results showing an immunoreactive band at 36 kDa in ALS samples, consistent with the apparent molecular size of FRZB (Fig. 1C). In contrast, there was only faint immunoreactivity in normal controls. Quantification by densitometry showed a nearly sixfold increase in ALS samples. Taken together, these findings show a significant increase of FRZB in ALS muscle samples compared to normal and myopathy controls. Neuropathy samples, although quite variable, trended higher compared to controls.

**Table 1:** Demographic and clinical data.

	Normal	ALS	Myopathy	Neuropathy
Number	28	43	13	13
Mean age^a^ (year)	58 ± 11	60 ± 12	53 ± 16	55 ± 11
Age range (year)	22–74	27–76	23–80	33–68
Gender (M:F)	1.5:1	1.4:1	1:2.3	2.3:1
Autopsy	9	11		
Biopsy	19	32	13	13
Duration^b^ (m)		19 ± 13		
Diagnosis	–	Spinal onset (33)	Inflammatory (8)	Non-specific axonal neuropathy^c^ (8)
		Bulbar onset (10)	Non-specific (3)	CIDP (3)
			Mitochondrial (1)	Plexopathy (1)
			Critical Illness (1)	Radiation induced (1)
**Muscles sampled**				
Biceps brachii	5	5	3	
Deltoid	6	7	3	3
Vastus lateralis	17	19	6	4
Tibialis anterior		12	1	6

*CIDP* chronic inflammatory demyelinating polyradiculoneuropathy, *SD* standard deviation.

^a^Mean age (± SD) at time of sample collection.

^b^Mean duration (± SD) of disease from onset of symptoms to time of sample collection. Duration of disease was unknown in two patients.

^c^No patients were diagnosed with Charcot Marie Tooth neuropathy.

### FRZB mRNA is elevated in the SOD1^G93A^ mouse early in the course of disease

**Figure 2:**
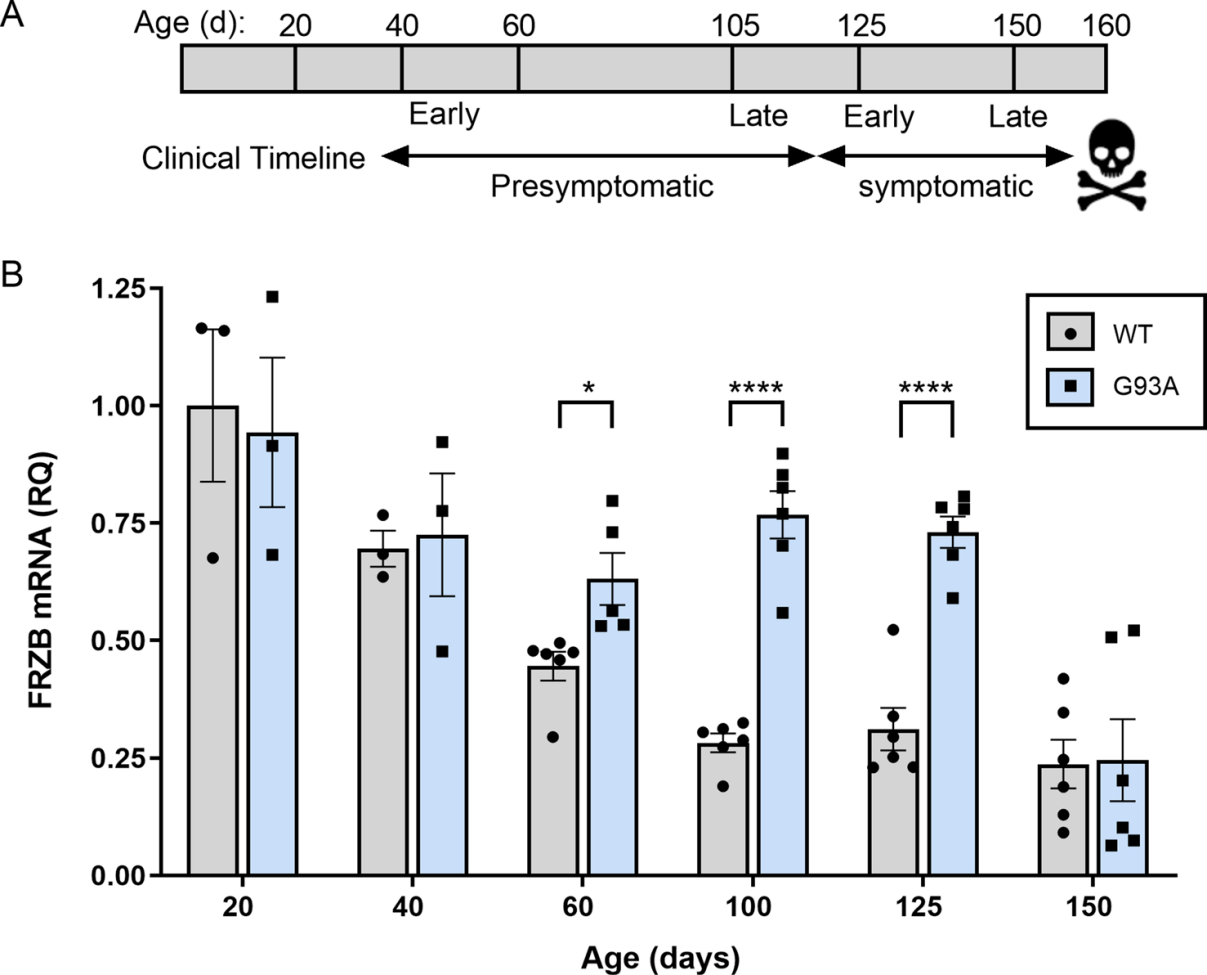
FRZB mRNA is upregulated in the gastrocnemius muscle of the SOD1^G93A^ mouse in presymptomatic and symptomatic stages of disease. (**A**) Time line of clinical progression in the SOD1^G93A^ mouse. (**B**) FRZB mRNA expression was quantitated relative to GAPDH endogenous control using qPCR at different ages in SOD1^G93A^ mice and littermate controls (WT). Mean FRZB expression in WT mice at 20 days was set to 1 and FRZB mRNA in all other groups was normalized to that group (Mean ± SD). For groups at 20 and 40 days, n = 3; 60, 100, 125, 150 days, n = 6. *P < 0.5; ****P < 0.0001.

Although patients with mutant SOD1 only represent up to 2% of the ALS population, the SOD1 mouse model reflects many of the clinicopathological changes that occur in ALS^16^. It provides an opportunity to examine temporal patterns in biomarker expression including disease onset and with disease progression. Here, we assessed the temporal pattern of FRZB mRNA in the SOD1^G93A^ mouse. This model is in the Bl6 background which has a longer lifespan than the original Bl6/SJL mouse^17^. We have previously used declines in rotarod performance and weight measurements to determine symptomatic onset^9^. Here, we sampled the gastrocnemius muscle at different timepoints reflective of this clinical timeline (Fig. 2A). In WT littermates, FRZB mRNA was highest at 20 days of age and showed a steady decline which leveled out by 100 days (Fig. 2B). In the SOD1^G93A^ mouse, however, FRZB mRNA did not decline, but remained significantly elevated beginning at 60 days (P < 0.05) compared to WT. This time frame is similar to what we previously observed with other muscle biomarkers in this model^8–11^. The fold-difference increased to ~ 2.5 through 125 days of age. At end-stage and just prior to death, the expression declined to WT levels. Taken together, FRZB appears to be developmentally regulated, but reflects ALS disease onset and remains elevated up until end-stage. BI, biceps brachii; DL, deltoid; TA, tibialis anterior; VL, vastus lateralis.

### FRZB is detected in ALS muscle myofibers

**Figure 3:**
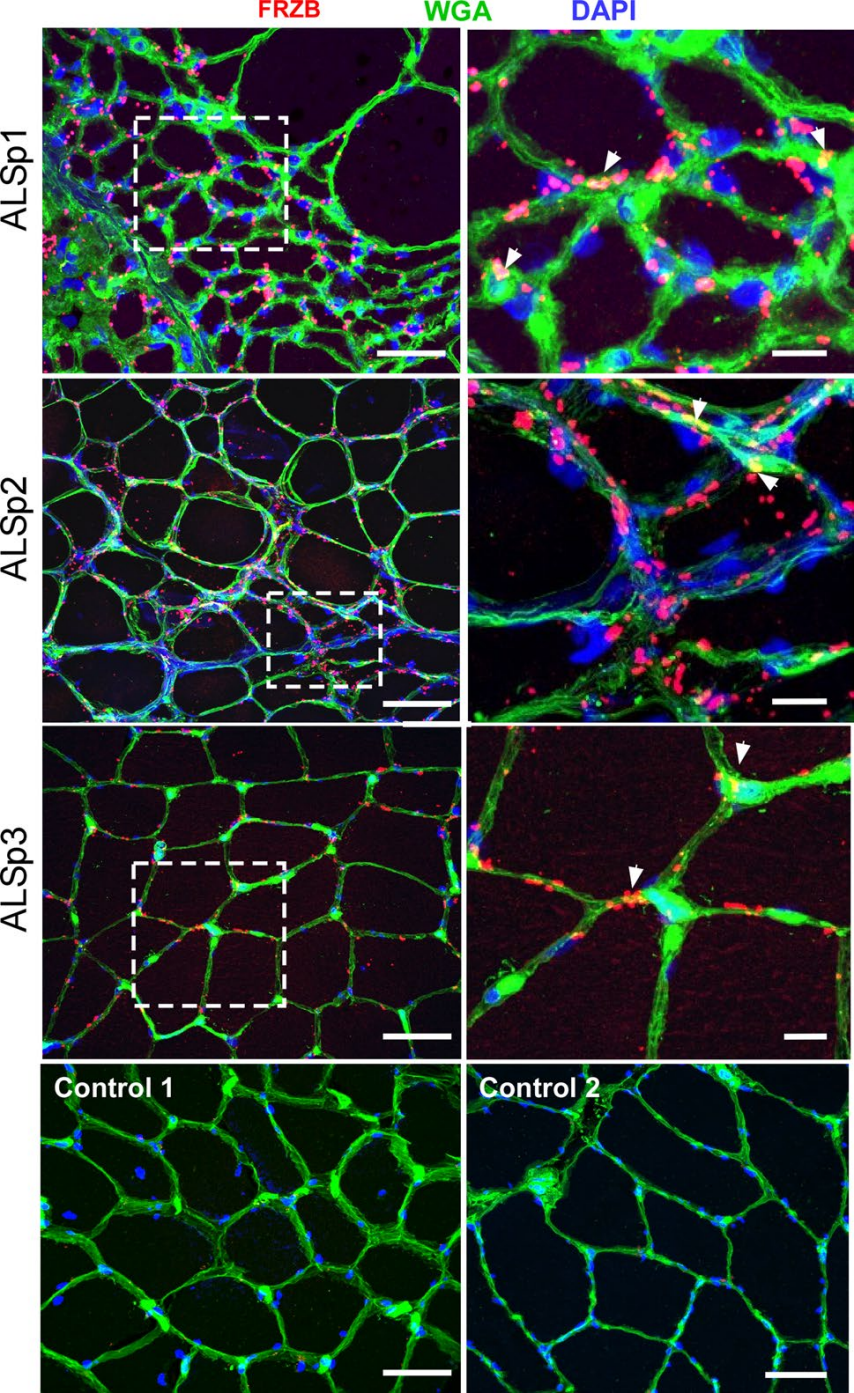
FRZB is upregulated in ALS myofibers. Immunohistochemistry of FRZB in vastus lateralis muscle in three ALS and two normal control subjects is shown. FRZB staining is punctate in appearance around the periphery of myofibers. Enlarged photomicrographs of boxed areas in the ALS sections are shown in the panels on the right. Arrowheads show areas of merged FRZB/WGA signal. No immunoreactivity is seen in two normal control muscle samples (bottom panels). Scale bars, 50 µM in low power views and 10 µM in the enlarged views.

**Figure 4:**
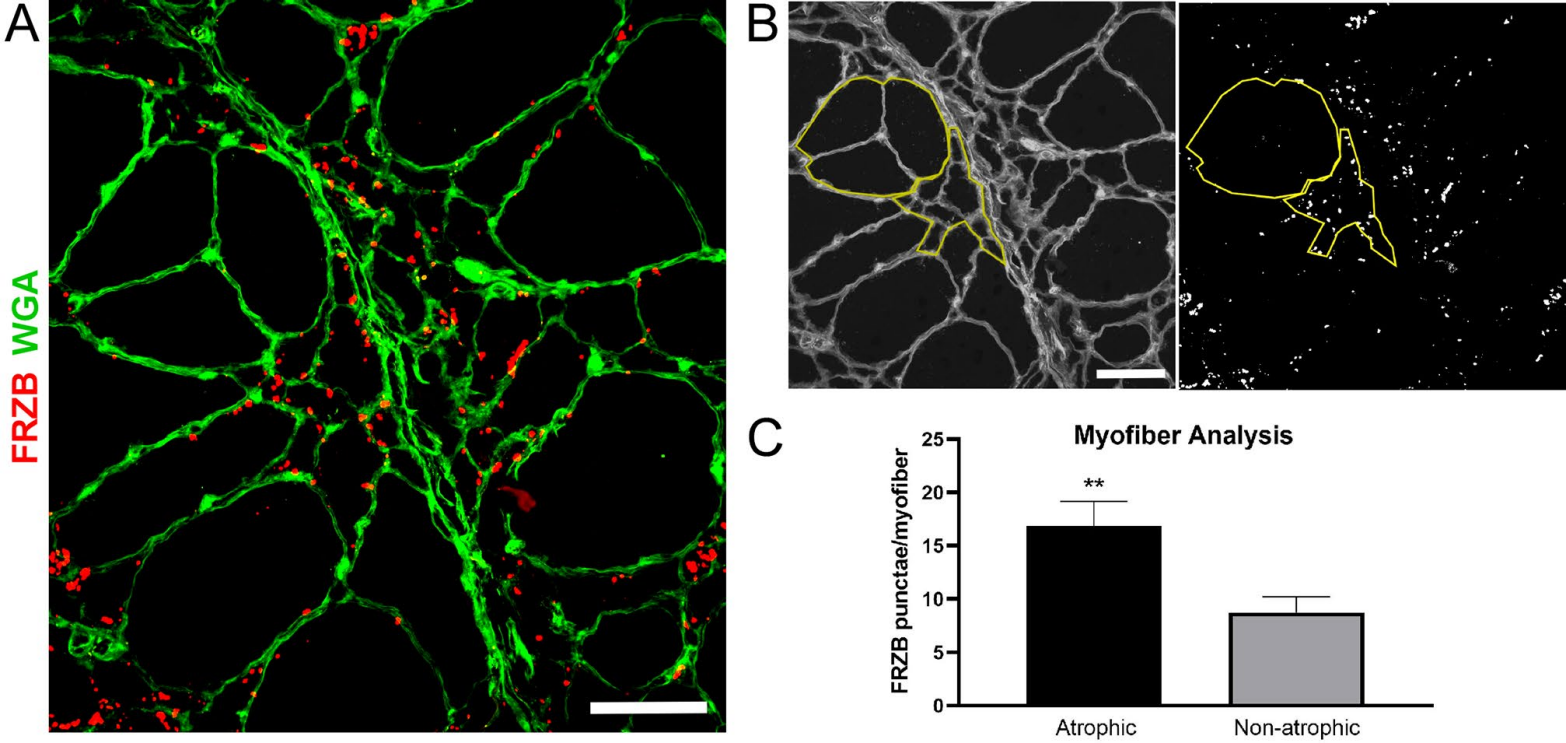
FRZB immunoreactivity is increased in atrophic myofibers of ALS muscle samples. Muscle sections from ALS patients were immunostained for quantification of FRZB punctae. (**A**) Representative section (from a deltoid muscle) showing the presence of both atrophic (< 25 µM) and non-atrophic myofibers (> 25 µM). The section is also immunostained with an FRZB antibody. (**B**) Micrographs were analyzed by ImageJ where the number of FRZB punctae associated with non-atrophic and atrophic myofibers was quantitated (yellow tracing shown in the representative section). (**C**) Quantitative results of FRZB-positive punctae in muscle sections from 5 ALS patients (1 deltoid, 1 biceps and 3 vastus lateralis muscles). Columns represent the mean ± SEM of 34 atrophic and 26 non-atrophic fibers analyzed. **P = 0.009. Scale bars, 50 μM.

**Figure 5:**
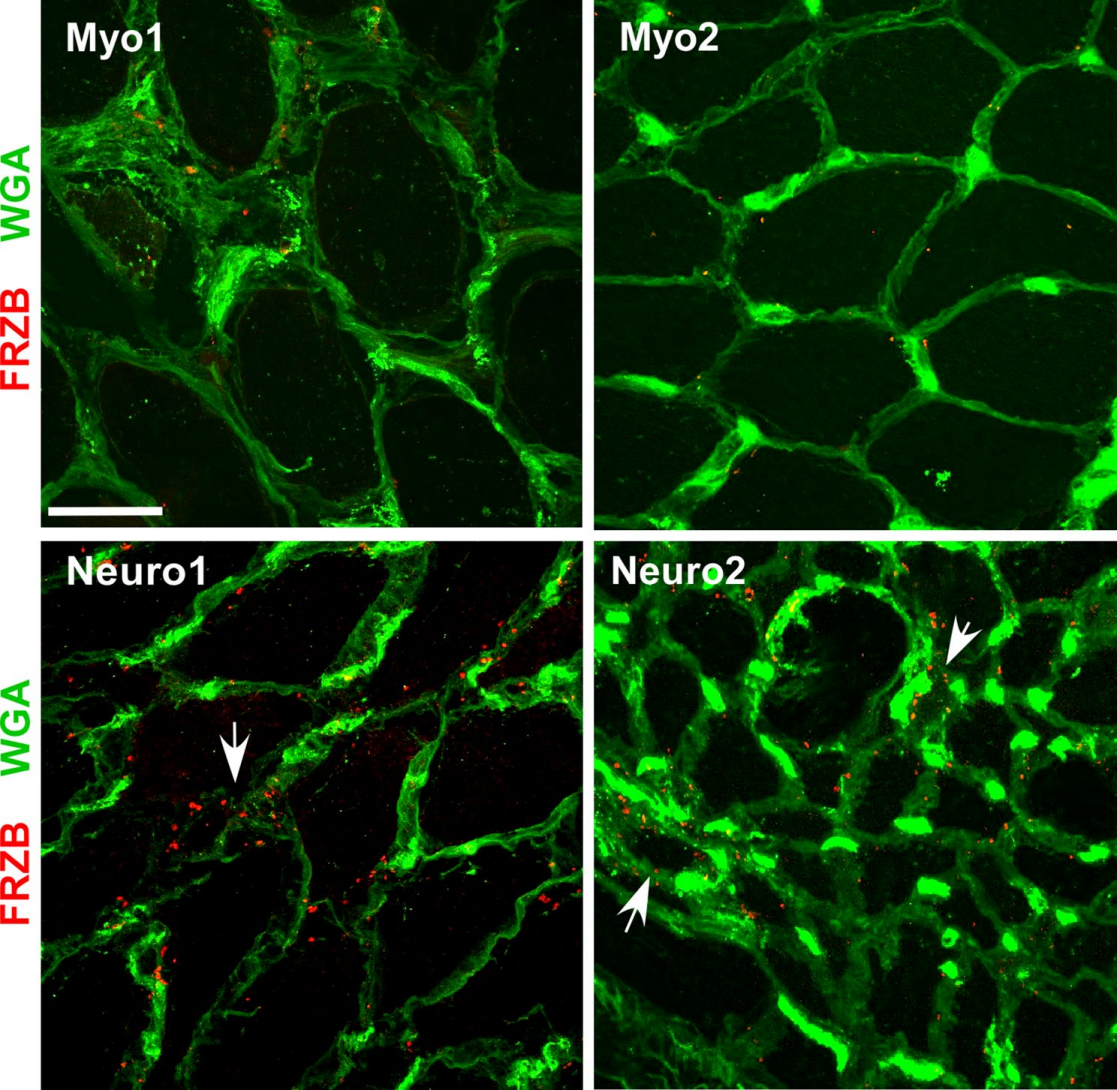
FRZB expression in disease controls. FRZB immunostaining comparing myopathy (Myo1, necrotizing myopathy, vastus lateralis; Myo2, inflammatory myopathy, biceps) and neuropathy (Neuro1, deltoid; and Neuro2, vastus lateralis) disease controls. Some immunostaining of FRZB is seen in the neuropathy controls surrounding myofibers that appear atrophied (arrows). Minimal to no punctate signal is seen in the myopathy samples. Scale bar, 50 µM.

Having validated the consistent upregulation of FRZB in muscle lysates of ALS and to a lesser extent non-ALS neurogenic disease, we performed immunohistochemistry to determine its location in muscle. In three ALS patient samples tested, we detected abundant FRZB immunoreactivity predominantly in the endomysial connective tissue (Fig. 3). The immunoreactivity was characterized by punctate foci surrounding myofibers and some colocalization with WGA. The punctae were variable in size, ranging from ~ 0.2 up to 8 μm. The larger punctae often appeared to be a coalescence of smaller punctae. Particularly intense staining was observed in areas of grouped atrophy, as characterized by large aggregations of small and angular fibers (ALSp1 and ALSp2). Immunoreactivity was also detected in areas where myofibers were more preserved, although to a lesser extent (ALSp3). No immunoreactivity was detected in muscle sections of normal subjects (Control 1 and Control 2). Additional ALS patient muscle sections were examined showing a similar pattern of punctate FRZB immunostaining with an increase in areas of myofiber atrophy either in groups or in isolation (Supplemental Fig. S2). To explore further the potential association of FRZB immunoreactivity with atrophic fibers, we quantified FRZB punctae associated with atrophic and non-atrophic fibers. A representative section is shown in Fig. 4A where both atrophic and non-atrophic fibers are present. With ImageJ, we selected muscle fibers for FRZB quantitation in muscle sections from ALS patients (a tracing of the muscle section in Fig. 4A is shown in Fig. 4B). We then calculated the average number of associated punctae and found a twofold increase with atrophic fibers (P = 0.009) (Fig. 4C). We next looked at neuropathic and myopathic disease controls (Fig. 5). Trace FRZB immunoreactivity was seen in two patients with myopathy (Myo1, necrotizing myopathy) and inflammatory; Myo2, inflammatory myopathy). In two patients with neuropathy, immunostaining was seen in a pattern similar to ALS sections, albeit less prominent, and again with a predilection for atrophic myofibers. Taken together, FRZB immunoreactivity was observed consistently in ALS and non-ALS denervated muscle, localized mainly to the endomysial connective tissue and muscle membrane, with more intense staining in atrophic fibers. FRZB expression in myopathic disease was low, consistent with the ELISA data (Fig. 1B).

### β-catenin levels are increased in ALS muscle myofibers

**Figure 6:**
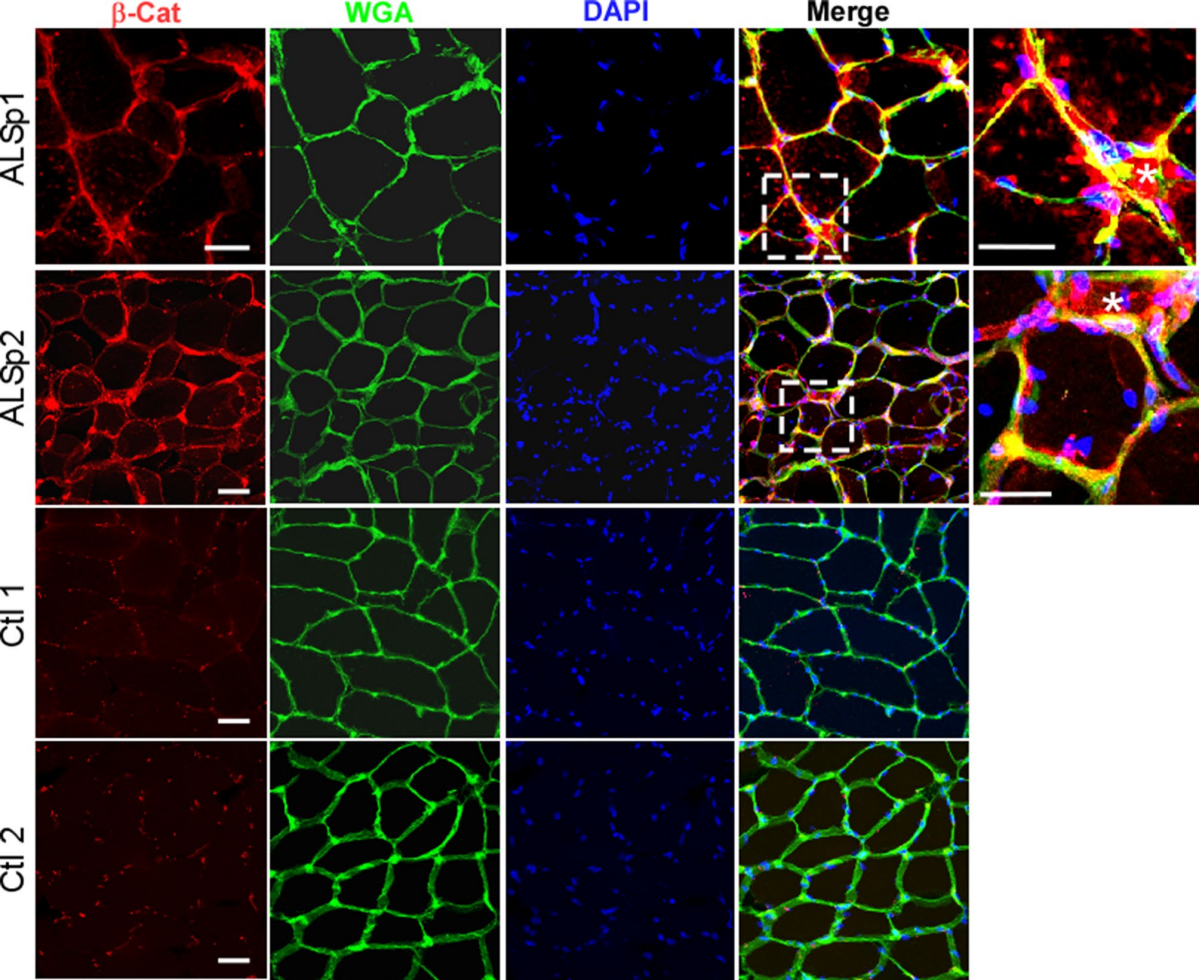
Increased levels of membranous β-catenin are detected in ALS muscle. Muscle sections from two ALS patients show increased β-catenin immunoreactivity which is predominantly membranous. Enlargement of the boxed area shows particularly intense myofiber staining of membrane associated β-catenin (merged signal with the membrane-associated lectin, WGA). Several of the myofibers are atrophic (asterisk). Sections from two age-matched healthy controls are shown below. Scale bars, 50 µM for low power views and 20 µM in the enlarged views (areas outlined by dashed boxes).

Since FRZB is a Wnt antagonist, we next looked at the canonical downstream Wnt signal transducer, β-catenin, by immunohistochemistry to assess its expression and localization. In muscle sections from two ALS patients, we observed intense β-catenin immunoreactivity predominantly localized to the muscle membrane as indicated by a merged signal with WGA (Fig. 6). As with FRZB, the signal intensity was high in areas of myofiber atrophy. In control muscle sections, there was punctate immunostaining which also appeared to be in proximity to the muscle membrane. Taken together, there were increased levels of membranous β-catenin detected in ALS muscle samples.

## Discussion

In this report, we validate the upregulation of FRZB, a gene first identified in a previous RNA sequencing project^9^, in skeletal muscle from a large cohort of ALS patients. This increase was paralleled by a concomitant increase in β-catenin levels predominantly at the muscle membrane. We show in the SOD1^G93A^ ALS mouse model that FRZB expression increased in the presymptomatic and symptomatic stages in parallel with disease progression. To a lesser extent, FRZB was also elevated in muscle samples from patients with other neurogenic disease, indicating that it is not specific for ALS. Whether it is a general marker of muscle denervation remains to be seen since there was modest detection of FRZB expression in myopathy samples. It is well recognized, however, that secondary denervation changes are frequently found in muscle biopsies of chronic and active inflammatory myopathies which comprised the majority of our samples^18^. Taken together, our findings identify FRZB as part of a molecular signature of muscle denervation in ALS that has the potential to track disease progression and to provide insight into molecular mechanisms of muscle denervation/reinnervation.

FRZB is a secreted protein that plays an important role in development, including myogenesis, bone and joint formation, and cartilage homeostasis^19–23^. Aberrant changes in FRZB expression have been associated with pathophysiological states including osteoarthritis and cancer (attenuated expression) and limb-girdle muscular dystrophy (increased expression)^23–25^. The role FRZB is playing in muscle denervation, whether mitigating or exacerbating, is unclear. FRZB and other putative Wnt antagonists participate in axon outgrowth and targeting in other neural systems^26,27^ and thus may come into play in a positive way since reinnervation involves motor neuron sprouting and reconnection to denervated NMJs. In ALS, the process of reinnervation is quite active, and animal studies suggest that denervation/reinnervation is continuous even within degenerating motor neurons^28,29^.

Wnt signaling plays an important function in the development and maturation of the NMJ and motor neuron termini^30–33^. Moreover, muscle-generated β-catenin is necessary for proper formation and function of motor neuron termini, and localizing motor axons for NMJ assembly^31,32,34^. Interestingly, a prior study observed a differential increase in Wnt ligand expression in the vicinity of NMJs of extraocular muscles (EOM) of ALS patients and the SOD1^G93A^ mouse^35^. The authors speculated that the relative sparing of EOM in ALS may be related to this differential upregulation. Thus, although FRZB is classically a Wnt antagonist, it may be working in concert with β-catenin in this context to promote reinnervation of NMJs in ALS.

In contrast to the potential salutary effects of Wnt signaling in reinnervation, it can promote fibrosis of muscle which is a pathological finding in ALS^31,36^. Prior reports have shown that TGF-β, a major driver of muscle fibrosis, requires β-catenin activation for its function^31^. Crosstalk between these two pathways is further underscored by the finding that TGF-β can stimulate β-catenin signaling^37^. We previously observed that all three TGF-β isoforms are abundantly expressed in ALS muscle samples and that this expression increases with disease progression^11^. Immunostaining patterns of TGF-β1 in that report were similar to FRZB here in that intense reactivity occurred in areas of myofiber atrophy. Thus, it is possible that activation of β-catenin is induced by TGF-β signaling and that FRZB is a compensatory response to attenuate this deleterious effect. Interestingly, in spinal muscle atrophy (SMA), another type of motor neuron disease, β-catenin signaling is also increased and its pharmacological inhibition improved NMJ pathology^38^. It should be stressed that additional studies are required to determine the level of β-catenin activation in ALS muscle such as its phosphorylation status, since its predominant localization was membranous rather than the nuclear (as seen with canonical Wnt signaling)^23^.

The utility of FRZB as a muscle biomarker in ALS is based on expression patterns in the SOD1^G93A^ ALS mouse (Fig. 4). Its upregulation started between 40 and 60 days of age which is a very early pre-clinical stage in this model, suggesting that it may be a marker of disease onset. This time frame is in keeping with the pattern we observed for a number of muscle biomarkers in ALS including Smads 1, 2, 3, 5, 8, all three isoforms of TGF-β, CYP27B1 and select myomiRs^8–11^. Taken together, these findings suggest a synchronized molecular response in skeletal muscle at or near the onset of disease in the SOD1^G93A^ mouse and prior to clinical symptoms determined by rotarod testing and weight loss^9^. Although the lack of specificity of FRZB for ALS-related muscle denervation may limit its use for diagnostic purposes, its relative increase in the SOD1^G93A^ mouse over different disease stages suggests that it reflects disease progression in the late pre-symptomatic and symptomatic stages. Neurogenic atrophy is a nearly uniform pathological finding in ALS^39^, and this reflects underlying motor neuron degeneration. Thus, the significant association of FRZB immunoreactivity with atrophic fibers supports its potential role for tracking disease progression. The increase in FRZB with other denervating disease is not unexpected as there are likely many shared pathways that become activated. Some components of the molecular signature that we have characterized previously, such as CYP27B1 and certain miRNAs, are also not specific for ALS^8,10^. While FRZB is a secreted protein, we could not detect increases in plasma samples from ALS patients (not shown). This could stem from limitations of the ELISA assay, but also suggests that FRZB remains local in the muscle microenvironment. The punctated and often coalesced immunostaining pattern raises the possibility that it is somehow sequestered or aggregated in the endomysial connective tissue. The origin of FRZB, however, cannot be discerned. Interestingly, the relative increase of FRZB mRNA in the ALS mouse muscle was related to a progressive decline of FRZB in control muscles with aging which suggests that FRZB is developmentally regulated. This may tie in with the potential role of FRZB in maturation of muscle innervation. Our work raises the possibility that β-catenin could also be a muscle biomarker of ALS. This gene was not identified in our original RNA sequencing analysis, so its increase may be due to changes in proteasomal degradation as seen in SMA. Additional studies will be required to validate its increase across a broader sampling of ALS muscles^38^.

Our work underscores the importance of Wnt signaling in ALS. Prior reports indicate upregulation of β-catenin, Wnt ligands and certain Wnt antagonists in astrocytes and neurons of spinal cords from human ALS subjects and the SOD1^G93A^ mouse^40–42^. Our report is the first to describe a consistent induction of FRZB in limb muscles of ALS patients, which in combination with elevated β-catenin expression, reflects the complexity of molecular responses in muscle denervation. It opens up avenues for future studies to functionally characterize these pathways in the context of ALS and to determine their potential as pharmacologic targets.

## Methods

### Human tissue

Human ALS and control muscle tissue samples were obtained from the archive of muscle biopsies at the UAB Division of Neuromuscular Disease after receiving approval from the UAB Institutional Review Board. Autopsy muscle samples were obtained from consented UAB patients who were enrolled in an ALS tissue collection program directed by PHK. The consent form and protocol were approved by the UAB Institutional Review Board. Samples were collected 3–10 h after death. Control autopsy samples from patients with no known neuromuscular disease were obtained with the assistance of the UAB Tissue Procurement Program (also approved by the UAB Institutional Review Board). All ALS patients ended up falling into the category of definite ALS by the El Escorial criteria and most were cared for at our institution. Normal control muscle samples were collected from patients with non-specific muscle symptoms (e.g. pain) but interpreted as normal by a neuromuscular pathologist. Neuropathy and myopathy disease control samples were chosen based on available clinical and electrophysiologic information in addition to histological evidence (denervation or myopathic changes) as determined by the neuromuscular pathologist. For immunohistochemistry, muscle samples were embedded in a mixture of tragacanth gum/OCT and flash frozen in an isopentane bath over liquid nitrogen.

### Animals

All animal procedures were reviewed and approved by the UAB Institutional Animal Care and Use Committee in compliance with the National Research Council Guide for the Care and Use of Laboratory Animals. B6.Cg-Tg (SOD1^G93A^) 1 Gur/J male mice (The Jackson Laboratory) were bred with C57BL/6J females to generate hemizygous SOD1^G93A^ offspring with wild-type (WT) littermates. These mice have later clinical onset and prolonged survival compared to the original ALS model on the B6/SJL background^17^. After sacrifice by CO_2_ inhalation followed by cervical dislocation, gastrocnemius muscle tissue samples were collected from SOD1^G93A^ and WT littermate controls at post-natal day 20, 40, 60, 105, 125 and 150. These time points cover the full range of disease stages in the ALS mouse: early pre-symptomatic (20, 40 and 60 days), late pre-symptomatic (105 days), early symptomatic (125 days), and late symptomatic (150 days) based on rotarod and weight testing as previously described^9^.

### RNA and protein analysis

RNA isolation from frozen muscle samples was performed using the mirVana miRNA isolation kit (Invitrogen). One microgram of RNA was reverse-transcribed using the High-Capacity cDNA Reverse Transcription Kit (Applied Biosystems) and FRZB mRNA expression was quantified by Taqman real-time PCR (Applied Biosystems). GAPDH expression was used as internal control as described previously^9^. For protein analysis, frozen muscle was lysed in T-PER lysis buffer (ThermoFisher) and quantified using the Pierce BCA Assay Kit (ThermoFisher). The Human sFRP-3 DuoSet ELISA (R&D Systems) was used to measure FRZB protein concentration in 50 μg of lysate per well. For Western blot, 50 µg of protein lysate was electrophoresed through a 4–20% Mini-PROTEAN TGX gel (Bio-Rad). After transfer to a nitrocellulose membrane, FRZB was probed with FRP-3 antibody (B-5, Santa Cruz Biotechnology). Chemiluminescent signal was imaged using the Chemidoc Imaging System (Bio-Rad). The blot was then stripped, re-probed with GAPDH antibody (Cell Signaling), and re-imaged. Densitometry measurements were performed using the Image Lab Software (Bio-Rad).

### Immunohistochemistry

Frozen muscle tissue was cut into 10 µm sections and air-dried at room temperature for 20 min followed by fixation with Bouin’s solution (Sigma-Aldrich). Slides were incubated in 3% hydrogen peroxide for 10 min. After blocking, slides were incubated with FRP-3 antibody (B-5, Santa Cruz Biotechnology) overnight at 4 °C. After washing in PBS, slides were incubated with Alexafluor-555 goat anti-mouse IgG (Invitrogen) secondary antibody. Sections were incubated in Wheat Germ Agglutinin (WGA), Oregon Green 488 Conjugate (ThermoFisher). Slides were then washed in PBS for a final time and cover slipped using Prolong Diamond Antifade Mountant with DAPI (Invitrogen). Slides were imaged using a Nikon C2 confocal microscope. For correlation of immunoreactive FRZB punctae with myofiber size, we used threshold-based analysis in ImageJ to quantify immunoreactive FRZB punctae in muscle sections from five ALS patients. Two high-powered fields per patient were analyzed. Myofiber size was determined using the minimal Feret’s diameter^43^. A myofiber diameter less than 25 μm was considered atrophic.

### Statistics

All statistical analyses were performed in Graphpad Prism 8. For human qPCR and ELISA, a Kruskal–Wallis one-way analysis of variance with Dunn’s multiple comparisons test was performed. A student’s T-test was performed for mouse qPCR data.

## Supplementary information

Supplementary Information.
