# Supplementary material for: Wnt antagonist FRZB is a muscle biomarker of denervation atrophy in amyotrophic lateral sclerosis
Source: Sci Rep. 2020 Oct 7;10:16679. doi: 10.1038/s41598-020-73845-z (PMC7541525; doi:10.1038/s41598-020-73845-z)
Supplement: Supplementary file 1 — Supplementary Information. [file 41598_2020_73845_MOESM1_ESM.pdf]

Supplemental Figures

Wnt antagonist FRZB is a muscle biomarker of denervation atrophy in amyotrophic lateral sclerosis

Thaddaeus Kwan, PhD, Mohamed Kazamel, MD, Kristina Thoenes, Ying Si, PhD, Nan Jiang, MD, PhD, Peter H. King, MD

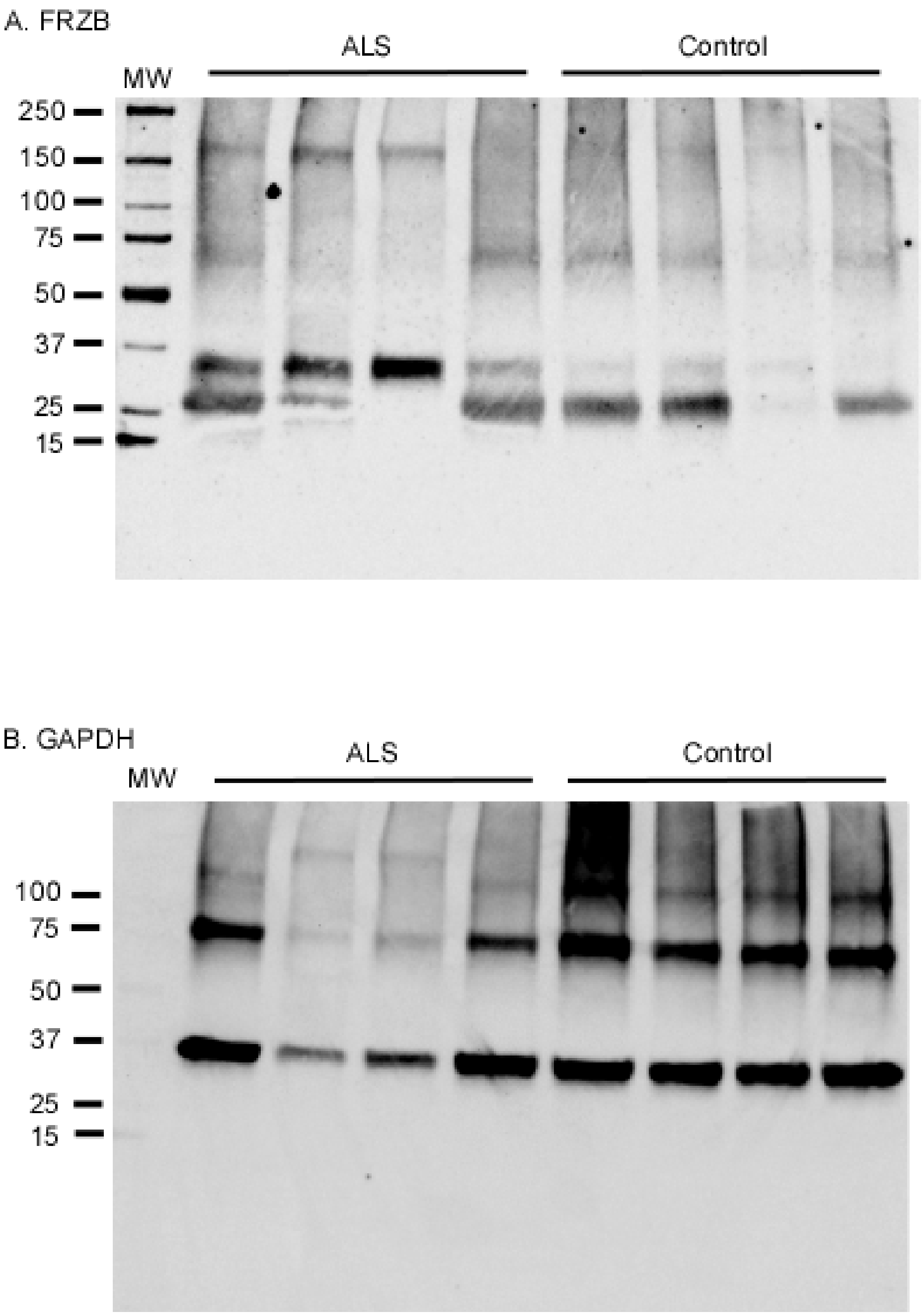

Supplemental Figure 1: Uncropped western blots for FRZB and GAPDH immunodetection shown in Fig. 1C.

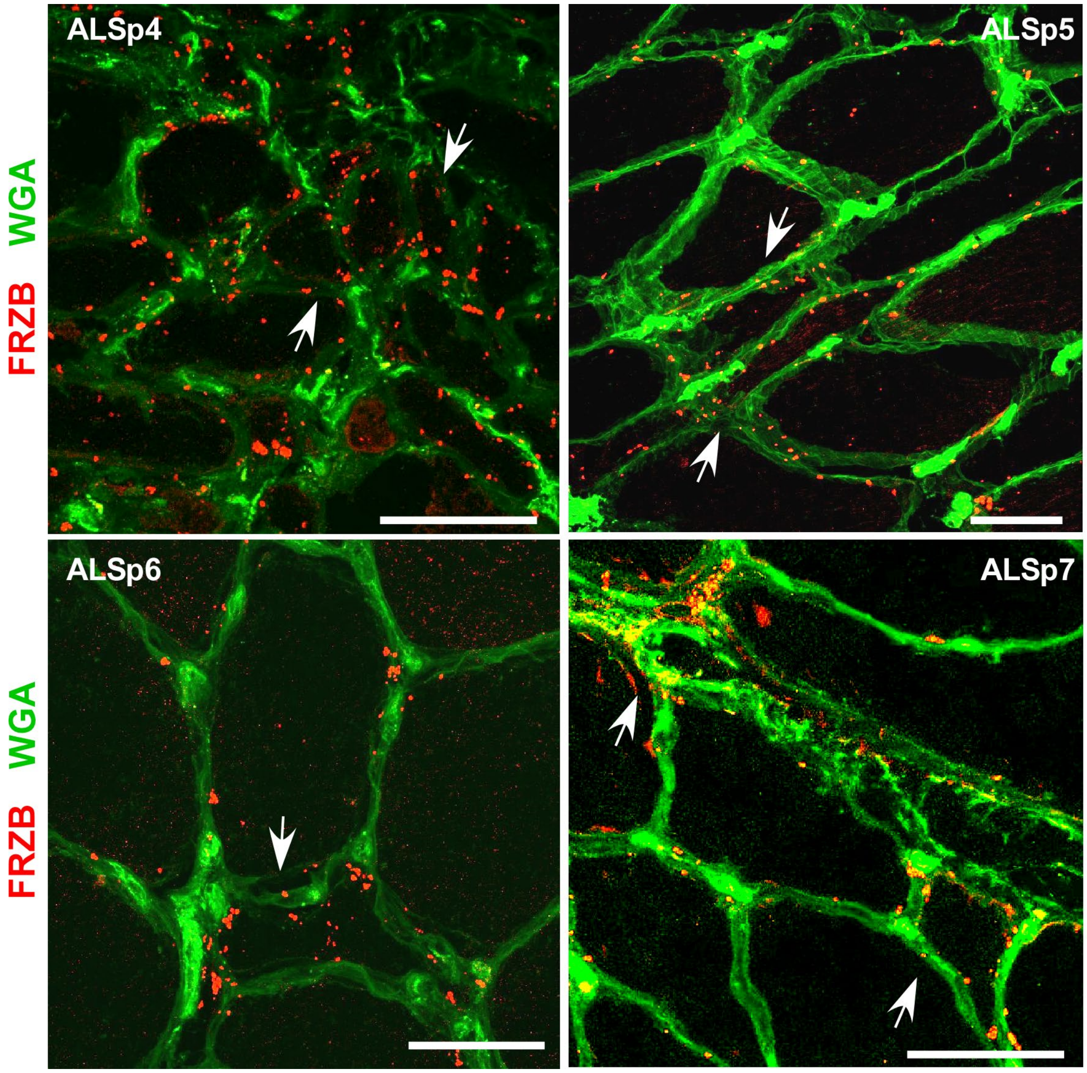

Supplemental Figure 2: FRZB is increased in areas of myofiber atrophy in ALS muscle samples. Muscle sections from four additional ALS patients were immunostained with FRZB antibody and the lectin, WGA. ALSp4 shows intense staining in a region of grouped atrophy (arrows). ALSp5-7 sections show increased immunoreactivity surrounding atrophic myofibers (arrows). Scale bars, 50 μM.
